# Supplementary material for: Glycated albumin as a diagnostic tool in diabetes: An alternative or an additional test?
Source: PLoS One. 2019 Dec 31;14(12):e0227065. doi: 10.1371/journal.pone.0227065 (PMC6938306; doi:10.1371/journal.pone.0227065)
Supplement: S1 Table — a Correlation is significant at the 0.01 level (2-tailed). b Correlation is significant at the 0.05 level (2-tailed). GA, glycated albumin; HbA1c, glycated haemoglobin; FPG, fasting plasma glucose; 2hPG, plasma glucose 2 h after oral glucose; BMI, body mass index; WC, waist circumference (cm); Trigl., Triglyceride; HDL, serum high density lipoprotein cholesterol; LDL, serum low density lipoprotein cholesterol. (DOCX) [file pone.0227065.s001.docx]

**S1 Table.** Correlations of GA, HbA_1c_ and factors potentially associated with the measurement of serum GA in all participants

|  | GA | HbA_1c_ | Age | BMI | WC | Albumin | Total cholesterol | Trigl. | HDL | LDL |
| --- | --- | --- | --- | --- | --- | --- | --- | --- | --- | --- |
| GA | 1 | 0.304^a^ | 0.294^a^ | -0.081 | -0.115 | -0.259^a^ | -0.049 | -0.197^a^ | 0.085 | -0.099 |
| HbA_1c_ |  | 1 | 0.267^a^ | 0.275^a^ | 0.240^a^ | -0.020 | 0.290^a^ | 0.148^b^ | 0.099 | 0.252^a^ |
| Age |  |  | 1 | -0.029 | 0.011 | -0.038 | -0.013 | 0.082 | 0.087 | -0.038 |
| BMI |  |  |  | 1 | 0.844^a^ | -0.079 | 0.122 | 0.130^b^ | -0.035 | 0.161^b^ |
| WC |  |  |  |  | 1 | -0.047 | 0.053 | 0.149^b^ | -0.057 | 0.090 |
| Albumin |  |  |  |  |  | 1 | 0.188^a^ | 0.177^a^ | -0.053 | 0.205^a^ |
| Total Cholesterol |  |  |  |  |  |  | 1 | 0.336^a^ | 0.131^b^ | 0.939^a^ |
| Trigl. |  |  |  |  |  |  |  | 1 | -0.446^a^ | 0.467^a^ |
| HDL |  |  |  |  |  |  |  |  | 1 | -0.175^a^ |
| LDL |  |  |  |  |  |  |  |  |  | 1 |

^a^ Correlation is significant at the 0.01 level (2-tailed). ^b^ Correlation is significant at the 0.05 level (2-tailed).

GA, glycated albumin; HbA_1c_, glycated haemoglobin; FPG, fasting plasma glucose; 2hPG, plasma glucose 2 h after oral glucose; BMI, body mass index; WC, waist circumference (cm); Trigl., Triglyceride; HDL, serum high density lipoprotein cholesterol; LDL, serum low density lipoprotein cholesterol.
